# Asbestos-related pleural and lung fibrosis in patients with retroperitoneal fibrosis

**DOI:** 10.1186/1750-1172-3-29

**Published:** 2008-11-13

**Authors:** Toomas Uibu, Ritva Järvenpää, Jari Hakomäki, Anssi Auvinen, Eero Honkanen, Kaj Metsärinne, Pekka Roto, Heikki Saha, Jukka Uitti, Panu Oksa

**Affiliations:** 1 Departments of Respiratory Medicine, Radiology, and Internal Medicine, Tampere University Hospital, Tampere, Finland; 2 Tampere School of Public Health, University of Tampere, Tampere, Finland; 3 Department of Medicine, Division of Nephrology, Helsinki University Central Hospital, Helsinki, Finland; 4 Department of Internal Medicine, Turku University Hospital, Turku, Finland; 5 Finnish Health Centers LTD, Tampere, Finland; 6 Clinic of Occupational Medicine, Tampere University Hospital and Finnish Institute of Occupational Health Tampere, Finland

## Abstract

**Background:**

Retroperitoneal fibrosis (RPF) is a rare fibroinflammatory disease that leads to hydronephrosis and renal failure. In a case-control study, we have recently shown that asbestos exposure was the most important risk factor for RPF in the Finnish population. The aim of this study was to evaluate the relation of asbestos exposure to radiologically confirmed lung and pleural fibrosis among patients with RPF.

**Methods:**

Chest high-resolution computed tomography (HRCT) was performed on 16 unexposed and 22 asbestos-exposed RPF patients and 18 asbestos-exposed controls. Parietal pleural plaques (PPP), diffuse pleural thickening (DPT) and parenchymal fibrosis were scored separately.

**Results:**

Most of the asbestos-exposed RPF patients and half of the asbestos-exposed controls had bilateral PPP, but only a few had lung fibrosis. Minor bilateral plaques were detected in two of the unexposed RPF patients, and none had lung fibrosis. DPT was most frequent and thickest in the asbestos-exposed RPF-patients. In three asbestos-exposed patients with RPF we observed exceptionally large pleural masses that were located anteriorly in the pleural space and continued into the anterior mediastinum.

Asbestos exposure was associated with DPT in comparisons between RPF patients and controls (case-control analysis) as well as among RPF patients (case-case analysis).

**Conclusion:**

The most distinctive feature of the asbestos-exposed RPF patients was a thick DPT. An asbestos-related pleural finding was common in the asbestos-exposed RPF patients, but only a few of these patients had parenchymal lung fibrosis. RPF without asbestos exposure was not associated with pleural or lung fibrosis. The findings suggest a shared etiology for RPF and pleural fibrosis and furthermore possibly a similar pathogenetic mechanisms.

## Background

Retroperitoneal fibrosis (RPF), or Ormond's disease, is a rare condition with fibrosis covering the abdominal aorta and the ureters. The etiology of RPF is generally unknown. It has been proposed that approximately one-third of RPF cases develop secondarily to aortic aneurysm, abdominal infections or surgery and as a side effect of several drugs, especially methysergide and other ergot derivates [1-3]. Asbestos is known to cause diffuse pleural thickening (DPT) and parietal pleural plaques [4]. High-level asbestos exposure may lead to the development of clinically detectable lung fibrosis (asbestosis) [5]. We have recently shown that asbestos exposure is one of the most important single risk factors for RPF, accounting for approximately 20% of all RPF cases in the Finnish population [6,7]. The aim of this study was to determine whether RPF patients have pleural or lung fibrosis and to assess the relations between asbestos exposure and intrathoracic fibrotic changes in RPF patients. Furthermore we evaluated the susceptibility for pleural and lung fibrosis among asbestos-exposed RPF patients and asbestos-exposed controls.

## Subjects and methods

### Study population

This material was part of our case-control study including 43 persons with RPF and 179 randomly assigned controls matched for year of birth, gender and central hospital district in Finland [6]. The diagnosis of RPF required the presence of the typical clinical condition–fibrosing mass covering the abdominal aorta and other retroperitoneal structures–and either histological confirmation (35 of 43 persons) or a follow-up of at least 1 year in order to rule out retroperitoneal malignancies (8 of 43 persons).

All of the participants were interviewed for medical history and asbestos exposure. The cumulative exposure to asbestos dust was estimated using fiber-years (40-hour shift per week at an average dust level of 1 fiber/ml for 1 year) and graded as follows: no significant asbestos exposure; slight exposure (asbestos exposure <10 fiber-years) and moderate-to-high exposure (asbestos exposure ≥ 10 fiber-years). Exposure was assessed by an occupational health physician with special expertise in the evaluation of asbestos exposure, the physician was blinded in terms of the case-control status of the participants. The details of the data collection have been given in our previous report [6].

We asked all of the unexposed and exposed patients with RPF and the controls with moderate-to-high asbestos exposure to participate in a study evaluating pleural and lung fibrosis with chest high-resolution computed tomography (HRCT). The Ethics Committee of the Tampere University Hospital approved the study protocol.

### Participation rate and demographic features

Altogether 38 (88%) of the patients with RPF and 18 (86%) of the asbestos-exposed controls were willing to participate. None of the 5 RPF patients who refused to participate in the HRCT study had notable asbestos exposure, which was also the main reason for their refusal.

The mean time since first asbestos exposure was 41.4 (SD 12.1) years for the RPF patients and 42.4 (8.9) years for the controls (Table 1).

**Table 1:** Demographic and exposure characteristics of the patients with retroperitoneal fibrosis and asbestos-exposed controls.

RPF	Asbestos exposure	Gender men/woman	Age		Pack-years of smoking		Age at diagnosis of RPF	
					
			mean	SD	mean	SD	mean	SD
Yes	No	9/7	61.9	9.7	20.4	18.8	55.5	9.6
Yes	Yes	19/3	64.1	9.4	27.0	17.4	54.9	8.0
No	Yes	18/0	66.0	7.7	22.4	27.1	NA	

(RPF = retroperitoneal fibrosis, NA = not applicable)

### Imaging

The HRCT was carried out in seven central hospitals. The HRCT scans consisted of 1-mm slices at 20-mm intervals from the first rib to the costophrenic angle in the prone position and with full inspiration. No contrast medium was used. The images were printed at two separate settings appropriate for viewing the lung parenchyma or the mediastinum and the pleura, the settings depending on the scanner used.

### Image analysis

All of the images were reviewed by two experienced thoracic radiologists. The reviewers were blinded to all medical information except the participants' names and identification numbers, which were printed on the films. The images were scored by consensus reading. Lung fibrosis, parietal pleural plaques and diffuse pleural thickening (DPT) were scored separately (additional files 1 and 2). The scoring was modified from our earlier classification systems [8,9]. Model images were not used, and the scoring was carried out in two sessions within one week. Measurements of the maximum DPT thickness were performed subsequently in one session. A definitely abnormal finding that could be related to asbestos exposure was rated class 1 for DPT (unilateral DPT <5 mm) and class 2 for pleural plaques (bilateral plaques on less than half of the slices) and lung fibrosis (at least 2 abnormal findings on both sides in several slices) (additional files 1 and 2).

### Classification of the pleural abnormalities

Pleural plaques are discrete areas of fibrous tissue limited to the parietal pleura, whereas diffuse pleural thickening or visceral pleural fibrosis is much more widespread and usually extends into the costophrenic angles [10-12]. Pleural plaques were diagnosed as sharply defined thickenings located internally with respect to a visible rib segment in the chest walls, paravertebral regions, or on the diaphragmatic surfaces, with or without calcification. Pleural thickening was classified as DPT if it appeared as a smooth, uninterrupted density with ill-defined margins and with extension of more than one-fourth of the pleural surface. Parenchymal bands extending from the pleural thickening to the lung parenchyma, rounded atelectasis, and the involvement of the interlobar fissures was used to differentiate DPT from pleural plaques. Rounded atelectasis was defined as a round or oval mass abutting the pleural surface and associated with the curving of pulmonary vessels or bronchi into the edge of the lesion [11]. The maximum thickness of the DPT was measured from the slices transversal with the thoracic wall.

### Statistical analysis

For the statistical analysis we combined the two asbestos-exposed RPF patient groups. The groups were compared using the Kruskal-Wallis and the Mann-Whitney tests, as appropriate. An ordinal logistic regression analysis was performed to assess the risk factors for DPT, pleural plaques and lung fibrosis in the asbestos-exposed patients and controls. The factors evaluated were the presence of RPF, age at the time of the HRCT, smoking in pack years, and the pleural plaque, DPT and lung fibrosis grade, as appropriate. The analysis was based on proportional odds (i.e. constant odds ratio across ordered categories of the response variable: odds of having a diagnostic score × or higher relative to having a score below ×). The outcome variable was the radiological finding categorized into four classes. The results of the ordinal logistic regression analysis therefore indicated susceptibility to the development of fibrotic changes, given asbestos exposure. Statistical significance was assessed using the likelihood ratio test. In addition, the susceptibility for asbestos-related pleural fibrosis among the RPF patients was evaluated in a case-case setting [13] using logistic regression analysis. All of the calculations were carried out with STATA 8.0 software (Stata Corporation, College station, TX, USA)

## Results

### Parietal pleural plaques

**Figure 1:**
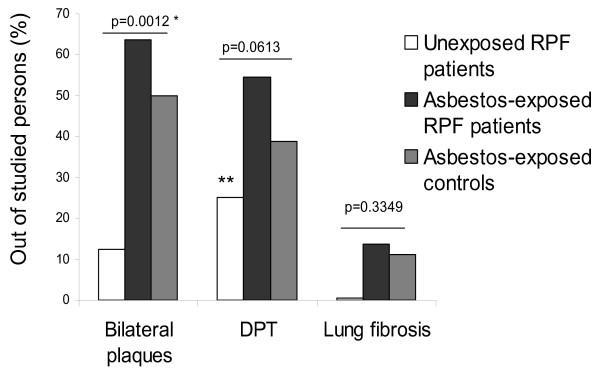
**Diffuse pleural thickening, pleural plaques and lung fibrosis**. Proportion of persons with diffuse pleural thickening (DPT) (class ≥ 1), bilateral parietal pleural plaques and lung fibrosis (class ≥ 2) among the unexposed patients with retroperitoneal fibrosis (RPF), the asbestos-exposed patients with RPF and the asbestos-exposed controls. * Difference between three groups, ** Difference between the unexposed and exposed RPF patients P = 0.045.

The unexposed RPF patients had only minor pleural plaques (≤ class 2), and the differences between this group and the asbestos-exposed groups were statistically significant (Figure 1, Table 2).

**Table 2:** Parietal pleural plaques, diffuse pleural thickening and lung fibrosis in the patients with retroperitoneal fibrosis (RPF) and the asbestos-exposed controls.

Radiological finding	RPF without asbestos exposure	RPF with asbestos exposure		Controls with ≥ 10 fy of asbestos exposure
			
	N = 16 % (N)	<10 fy N = 13 % (N)	≥ 10 fy N = 9 % (N)	N = 18 % (N)
Pleural plaques				
class 0	68.8 (11)	23.1 (3)	11.1 (1)	22.2 (4)
class 1	19.8 (3)	15.4 (2)	22.2 (2)	27.8 (5)
class 2	12.5 (2)	38.5 (5)	33.3 (3)	27.8 (5)
class 3–5	0 (0)	23.1 (3)	33.3 (3)	22.2 (4)
DPT				
class 0	75.0 (12)	46.2 (6)	44.4 (4)	61.1 (11)
class 1	6.3 (1)	0 (0)	11.1 (1)	11.1 (2)
class 2	18.8 (3)	23.1 (3)	0 (0)	33.3 (3)
class 3	0 (0)	30.8 (4)	44.4 (4)	22.2 (2)
Lung fibrosis				
class 0	87.5 (14)	69.2 (9)	66.7 (6)	77.8 (14)
class 1	12.5 (2)	15.4 (2)	22.2 (2)	11.1 (2)
class 2	0 (0)	7.7 (1)	0 (0)	0 (0)
class 3–5	0 (0)	7.7 (1)	11.1 (1)	11.1 (2)

(fy = fiber years, DPT = diffuse pleural thickening)

More than 60% of the asbestos-exposed RPF patients and half of the exposed controls had bilateral pleural plaques (Figure 1, Table 2), and almost half of them had widespread plaques in class ≥ 3. The frequency and quantity of the pleural plaques were similar in both of the asbestos-exposed groups. There were no differences between the asbestos-exposed cases and controls with respect to susceptibility to the development of parietal pleural plaques in the ordinal logistic regression analysis. Out of the studied variables, only lung fibrosis was associated with parietal pleural plaques (OR 3.78, 95% CI 1.52–9.43). The grade of pleural plaques was not related to age, smoking history, or DPT grade (additional file 3). In the case-case analysis, the OR for pleural plaques related to asbestos was 12.2 (Table 3).

**Table 3:** Pleural fibrosis consisting of bilateral parietal pleural plaques (PPP) and diffuse pleural thickening (DPT) in the patients with retroperitoneal fibrosis (RPF pts) regarding their asbestos exposure

	Unexposed RPF pts	Asbestos-exposed RPF pts	OR (95% CI)
Pleural fibrosis -	10	5	
Pleural fibrosis +	6	17	5.7 (1.4 – 23.4)
PPP -	14	8	
PPP +	2	14	12.2 (2.2 – 68.2)
DPT -	12	10	
DPT +	4	12	3.6 (0.9 – 14.7)

(OR = Odds ratio)

### Diffuse pleural thickening

DPT occurred more frequently among the RPF patients with asbestos exposure than among the unexposed patients (P = 0.045). There were no differences between the asbestos-exposed RPF patients and controls (P = 0.190) and none of the differences between the three groups reached statistical significance (Figure 1, Table 2). Among the RPF patients, asbestos exposure increased the risk for both DPT and for all pleural fibrotic changes (Table 3).

**Figure 2:**
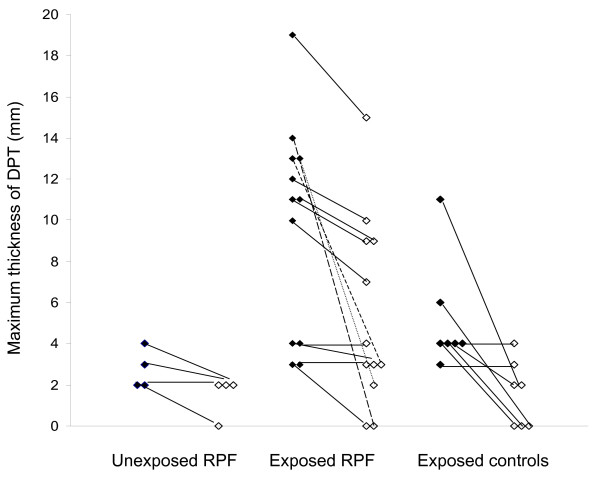
**Maximum thickness of diffuse pleural thickening**. Maximum thickness of diffuse pleural thickening (DPT, black diamonds), and the respective values for the contralateral pleura (white diamonds), in the unexposed patients with retroperitoneal fibrosis (RPF), the asbestos-exposed patients with retroperitoneal fibrosis and the asbestos-exposed controls. The lines connect each individual's values. "0" indicates that no diffuse pleural thickening was detected.

The mean maximum thickness of DPT was 2.8 (SD 1.0) mm for the 4 unexposed RPF patients, 9.8 (SD 5.1) mm for the 12 exposed patients with RPF and 5.1 (SD 2.7) mm for the 7 exposed controls (Figure 2). The difference between the three groups was significant (P = 0.040), and a similar difference was found for contralateral pleural thickening (P = 0.048). The respective values for the contralateral DPT were 2 (SD 0) mm (3 subjects), 6.5 (SD 4.2) mm (10 subjects), and 2.8 (SD 1.0) mm (4 subjects) (Figure 2).

Rounded atelectasis was detected in one unexposed RPF patient (6%), one asbestos-exposed control (6%) and five asbestos-exposed RPF patients (22%), three of whom had bilateral findings. No statistical difference was noted (p = 0.182).

In the ordinal logistic regression analysis, the asbestos-exposed RPF patients had a nonsignificantly increased risk for the development of DPT when compared with that of the asbestos-exposed controls (OR 3.06, 95% CI 0.81–11.56). Age at the time of the HRCT, smoking history, pleural plaques, and lung fibrosis grade had no influence on the development of DPT (additional file 3).

The four patients with RPF related to the previous use of ergotamine derivates had no DPT.

### Pleural masses

**Figure 3:**
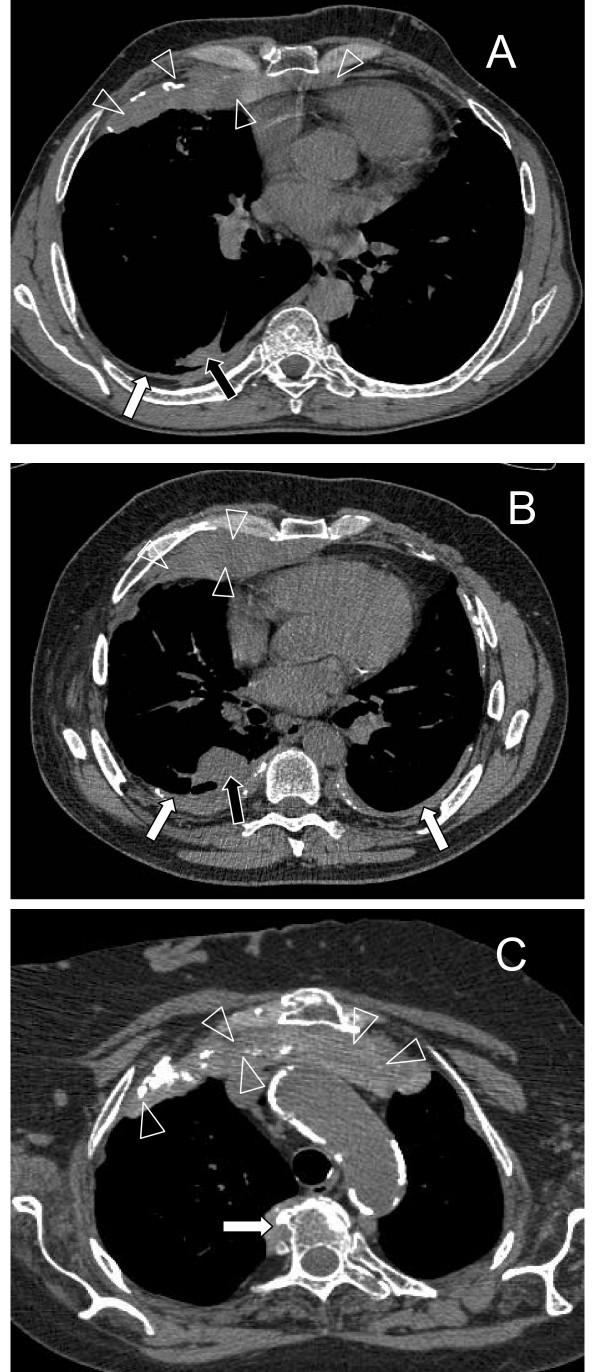
**Pleural masses in the patients with retroperitoneal fibrosis (RPF)**. A: High-resolution computed tomography scan of the lung of a 55-year-old former pipefitter with RPF; there is a large anterior pleural mass (maximum dimensions of 30 mm × 150 mm) continuing into the mediastinum (arrowheads) and a thinner dorsal diffuse pleural thickening (white arrow) with a rounded atelectasis (black arrow); the patient had undergone left-sided pleural decortication 10 years earlier. B: An anterior mediastinal mass with maximum dimensions of 30 mm in thickness and 190 mm in width (arrowheads), bilateral diffuse pleural thickening (white arrows) with a rounded atelectasis on the right side (black arrow); this 62-year-old RPF patient had worked as a storeman, had used asbestos gloves and sealing tapes and done some pipe insulation. C: A 76-year-old female with RPF worked as a construction cleaner and had had a high level of asbestos exposure; there is a large plaque-like mass with calcifications with maximum dimensions of 27 mm in thickness and 150 in mm width. A smaller paravertebral plaque (white arrow) has no continuity with retroperitoneal fibrosis.

We observed exceptionally large pleural masses in three asbestos-exposed patients with RPF. The uniform masses were located anteriorly in the pleural space and continued into the anterior mediastinum (Figure 3). The overall volumes of these masses clearly differed from the plaques and DPT found in the other persons. These unique fibrotic findings were omitted from the DPT thickness assessment, which was measured from the continuous dorsal fibrotic sheet.

### Lung fibrosis

There was no notable lung fibrosis in the unexposed RPF patients, but the three groups did not differ statistically in this respect (Figure 1, Table 2). One asbestos-exposed RPF patient had mild lung fibrosis (class 2), and two had moderate fibrotic changes (class 3), as did two controls. The exposed RPF patients were not more susceptible to lung fibrosis than the asbestos-exposed controls (OR 1.29, 95% CI 0.25–6.63). Lung fibrosis was associated with the occurrence of pleural plaques (OR 2.73, 95% CI 1.10–6.78). Age, smoking and DPT did not affect the development of lung fibrosis (additional file 3).

Most of the asbestos-exposed RPF patients with DPT also had bilateral pleural plaques and vice versa. One person had all three of the distinctive abnormal findings. The most typical findings of the asbestos-exposed controls were bilateral pleural plaques, and 1 subject from the control group had all three notable changes. Twelve (75%) of the unexposed RPF patients, six (27%) of the asbestos-exposed RPF patients and six (33%) of the asbestos-exposed controls did not have any of these changes.

### Pleural fibrosis at the time of the RPF diagnosis

Using patient's medical files, we were able to evaluate the presence of pleural fibrosis in 14 out of 17 RPF asbestos-exposed patients having fibrosis in the current HRCT. At the time of the RPF diagnosis, eight of them had had fibrotic changes in their thoracic X-ray and 6 had not. The subjects having pleural fibrosis at the time of the RPF diagnosis had a higher mean score for both PPP (2.8 versus 1.5) and DPT (2.1 versus 1.0) compared with the ones who had developed pleural changes after the appearance of RPF. These differences were not of statistical significance.

## Discussion

This study describes the association between RPF and asbestos-related lung diseases. We hope that our results help to identify persons who have developed RPF through occupational exposure to asbestos. Even though the findings of the current study alone are not enough to declare a causal association between asbestos exposure and RPF, they strengthen the validity of the results of our earlier case-control study and clarify the phenotype of asbestos-related RPF.

To our knowledge, the literature contains only three reports describing asbestos-related pleural findings in altogether five RPF patients [14-16]. In our study 16 out of 22 (73%) asbestos-exposed RPF patients had asbestos-related pleural pathology in their chest HRCT. The prevalence of pleural plaques, DPT and lung fibrosis found in the asbestos-exposed RPF patients was similar to that determined for the asbestos-exposed controls, but DPT was clearly more extensive in the asbestos-exposed RPF patients. Only a few RPF patients and controls with more than 10 fiber-years of asbestos exposure had asbestosis. It seems that the exposure level associated with the development of RPF is comparable to that associated with the development of pleural fibrosis rather than to the high level of exposure that induces asbestosis.

On the basis of our results, it can be argued that RPF is an independent risk factor for pleural fibrosis. The results of the case-control setting (ordinal regression analysis, additional file 3) indicate that RPF patients are more prone towards the development of severe DPT than exposed control subjects. The small number of cases did not allow us to evaluate the interactions between asbestos and RPF. Pleural fibrosis was evident at the time of the RPF diagnosis in most of the cases. Asbestos exposure occurs mainly via the respiratory system, and pleural fibrosis is far more common than RPF. It has been estimated that there are approximately 200 000 asbestos-exposed people [17], 80 000 men with bilateral pleural plaques, and even more with DPT [18] and, according to our estimations, 70–100 patients with RPF in Finland. Taking into consideration these findings, we suggest that asbestos-exposed subjects with RPF develop concomitant pleural fibrosis because of their higher individual susceptibility for asbestos-induced fibrosis.

Parietal pleural plaques are considered pathognomonic for asbestos exposure, and hence they also serve as an indicator of past exposure [19]. The clear difference between the unexposed and exposed groups with a positive trend in the RPF subgroups with slight and moderate exposure strengthens the validity of the results of our previous exposure risk assessment [6].

The percentage of bilateral pleural plaques in the asbestos-exposed groups was similar to those in previous studies, in which similar asbestos-exposed cohorts in Finland have been studied with CT scanning or autopsy [20,21]. Two of the eighteen patients with RPF but assumed to have had no asbestos exposure, had some bilateral plaques but no other evaluated abnormalities. This finding probably reflects a high urban background of amphibole asbestos anthophyllite, which was previously widely used in Finland and results in a relatively high prevalence of PPP in the Finnish urban population [21].

DPT was the most frequent among the asbestos-exposed RPF patients and it was thicker than in the asbestos-exposed controls or in the unexposed RPF patients. DPT is thought to be a consequence of acute asbestos-related pleurisy [22]. However, DPT is not specific to asbestos exposure and may also result from other inflammatory conditions, such as infections, trauma, surgery and drug reactions (eg to ergot derivates) [23]. Crocidolite-related DPT has been shown to progress in the first 15 years after its diagnosis [24], and this progression concurs with our clinical experience in Finland with the past use of amphibole asbestos. We think that the thinner DPT seen in unexposed RPF patients may be the result of short-lasting injury such as surgery or infection, and the thicker DPT found in asbestos-exposed persons is probably related to continuous irritation caused by bioresistant amphibole fibers.

Available CT scans of RPF tissue in asbestos-exposed patients show large unresolved masses that are probably, for the most part, acellular fibrous tissue resembling the one found in DPT.

DPT, unlike parietal pleural plaques, causes significant restrictive impairment of lung function [25,26]. The latency time for DPT is typically over 20 years from the beginning of asbestos exposure, although benign asbestos pleurisy can occur earlier [27]. DPT can be induced by moderate asbestos exposure, and the amount of exposure required for the development of DPT is probably higher than for parietal pleural plaques [28]. Nine out of the eleven asbestos-exposed patients with RPF and bilateral DPT also had bilateral pleural plaques (class ≥ 2). Marked DPT masks parietal plaques, and some patients with class 2 plaques and thick DPT may, in fact, have had bilateral plaques of class 3.

Ergot drugs have been shown to cause pleural effusion and DPT [23]. This finding is particularly interesting because the use of ergotamine derivates is also a well known risk factor for RPF [29]. In our study, however, the persons having RPF in relation to the use of ergoline medication had no signs of DPT. The pleural effusion and DPT induced by asbestos and ergot drugs share common features, and the etiological diagnosis is difficult for persons with both exposures [30].

Three asbestos-exposed RPF patients had exceptionally large anterior pleural masses extending into the anterior mediastinum. All of them also had typical asbestos-related findings: widespread bilateral plaques in all three; dorsal DPT in two cases (Figure 3A and 3B) and fibrotic lesions fulfilling the criteria for asbestosis in one case (Figure 3C, not shown with the parenchymal settings). In all of these cases the pleural masses were visible in the chest X-rays taken at the time of the diagnosis of RPF. The coexistence of large masses in the pleural and retroperitoneal space suggests a common etiology, although there was no continuity between the mediastinal and retroperitoneal masses. In our experience, such changes are rarely found even in asbestos-exposed persons having other marked pleural pathology. Two similar cases having slight asbestos exposure and no other known risk factors for RPF have been recently reported in France [16]. It seems that asbestos can induce unusually severe fibrotic reaction in some susceptible individuals.

Our study showed that the frequency of asbestos-related lung fibrosis in RPF patients was not higher than that of the asbestos-exposed controls. It has been widely accepted that the development of asbestosis requires high-level asbestos exposure, a minimum of 20–25 fiber-years [31]. Most of our patients and the controls had exposure of <20 fiber-years, and, therefore, the proportion of persons with asbestosis was low.

Although we propose that pleural and retroperitoneal fibrosis may both be caused by asbestos fibers, there are certain differences in the clinical picture of pleural fibrosis and RPF. RPF is usually symptomatic, causing poorly localized pain in the abdominal, flank, or back region. Symptoms and laboratory findings suggesting systemic inflammation–weight loss, fever and nausea, a clearly elevated erythrocyte sedimentation rate and anaemia–are frequently present [32]. DPT usually progresses slowly and is asymptomatic in many cases, and parietal plaques cause no symptoms. Only patients with acute asbestos pleurisy may have local and systemic symptoms and a moderately elevated erythrocyte sedimentation rate [27]. Corticosteroids usually have a dramatic effect on inflammation in RPF, and together with surgical management of ureteric obstruction are the mainstay treatment for RPF [33]. Corticosteroids have no role in the management of DPT, but may alleviate the symptoms of acute asbestos pleurisy.

Albeit our study population is one of the largest published sets of RPF patients in the literature, the numbers of participants in our study was still rather small. Therefore we combined the groups of RPF patients with slight and moderate-to-high asbestos exposure. This combined group of RPF patients had, on the average, less asbestos exposure than the control group with exposure of ≥ 10 fiber-years in all cases. The ordinal logistic regression modeling may, therefore, have underestimated the risk of pleural fibrosis in association with RPF.

On the basis of our epidemiologic work and our current study we propose the following criteria for the classification of RPF as an occupational disease: (i) occupational asbestos exposure of ≥ 10 fiber-years (OR 8.8) or (ii) occupational asbestos exposure of <10 fiber-years (OR 5.5) combined with bilateral pleural plaques or DPT or both pleural plaques and DPT. The presence of asbestosis (parenchymal fibrosis) should not to be required for the diagnosis of asbestos-related RPF. Asbestos-related RPF, like asbestos-related pleurisy, should be a diagnosis of exclusion. Nevertheless, asbestos-related pleural findings should be taken into account also in the presence of other risk factors, such as ergotamine medication or abdominal aortic aneurysm.

## Conclusion

In conclusion, the majority of the asbestos-exposed patients with RPF had asbestos-related pleural fibrosis and it was more extensive than in the asbestos-exposed controls. Lung fibrosis was equally frequent among the asbestos-exposed RPF patients and the controls. RPF without asbestos exposure had no association with pleural or lung fibrosis.

The findings suggest a shared etiology for RPF and pleural fibrosis and possibly similar pathogenetic mechanisms in some subjects. All RPF patients should be evaluated for asbestos exposure, and lung HRCT should be performed if appropriate.

## Competing interests

The authors declare that they have no competing interests.

## Authors' contributions

TU was responsible for the data collection, statistical analysis and preparation of the article; RJ and JH evaluated the CT scans; AA planned the data analysis; EH, KM and HS participated in the identification of the RPF patients. PR was the initiator of the study, JU acted as an expert consultant and PO evaluated the asbestos exposure. All of the investigators contributed to the study design and writing of the manuscript.

## Supplementary Material

Additional file 1 **Classification of the pleural findings**. Click here for file

Additional file 2 **Classification of lung fibrosis**. Click here for file

Additional file 3 **Adjusted odds ratios for potential risk factors for parietal pleural plaques, DPT and lung fibrosis in the asbestos-exposed subjects according to an ordinal regression analysis**. Click here for file
