# Supplementary material for: Asbestos-related pleural and lung fibrosis in patients with retroperitoneal fibrosis
Source: Orphanet J Rare Dis. 2008 Nov 13;3:29. doi: 10.1186/1750-1172-3-29 (PMC2596089; doi:10.1186/1750-1172-3-29)
Supplement: Additional file 1 — Classification of the pleural findings. [file 1750-1172-3-29-S1.doc]

### Additional file 1. Classification of the pleural findings.

| Class |  | Description |
| --- | --- | --- |
| Parietal pleural plaques |  |  |
| 0 |  | Normal finding |
| 1 |  | Subnormal finding, plaques suspected or unilateral plaques |
| 2 |  | Bilateral plaques on less than half of the slices |
| 3 |  | Bilateral plaques on at least half of the slices |
| 4 |  | Bilateral plaques on at least two-thirds of the slices |
| 5 |  | Bilateral plaques exceeding 50% of the total pleural area |
| Diffuse pleural thickening |  |  |
| 0 |  | Normal finding |
| 1 |  | Unilateral pleural thickening of <5 mm |
| 2 |  | Bilateral pleural thickening of <5 mm |
| 3 |  | Uni- or bilateral pleural thickening of ≥5 mm |
