# Supplementary material for: Asbestos-related pleural and lung fibrosis in patients with retroperitoneal fibrosis
Source: Orphanet J Rare Dis. 2008 Nov 13;3:29. doi: 10.1186/1750-1172-3-29 (PMC2596089; doi:10.1186/1750-1172-3-29)
Supplement: Additional file 2 — Classification of lung fibrosis. [file 1750-1172-3-29-S2.doc]

### Additional file 2. Classification of lung fibrosis.

| Assessed abnormalities* | | |  |  | |
| --- | --- | --- | --- | --- | --- |
| Subpleural nodules/ irregular opacities | | | | |  |
| Septal lines (5 per lung on a slice on at least 2 slices) | | | | |  |
| Curvilinear opacities on at least on 1 slice | | | | |  |
| Intralobular fibrosis on at least on 2 slices | | | | |  |
| Parenchymal bands on at least on 2 slices | | | | |  |
| Honeycombing | | | | |  |
|  |  |  | | |  |
| Fibrosis Classes |  | Description | | | |
| 0 Normal |  | Normal finding by all criteria | | | |
| 1 Subnormal |  | 1-2 criteria, no honeycombing | | | |
| 2 Mild fibrosis |  | At least 2 criteria on both sides in several slices, no honeycombing | | | |
| 3 Moderate fibrosis |  | Several criteria on at least 5 slices or extending deep into the lung; no honeycombing | | | |
| 4 Severe fibrosis |  | Several criteria as in class 3 and honeycombing; lung architectural change | | | |
| 5 Extreme fibrosis |  | Extremely severe and various fibrotic changes, little normally aerated lung left | | | |

* Abnormalities clearly associated with processes unrelated to asbestos-related diseases (eg, focal scarring and fibrosis related to centrilobular emphysema) were not included in the fibrosis scale.
