# Supplementary material for: Asbestos-related pleural and lung fibrosis in patients with retroperitoneal fibrosis
Source: Orphanet J Rare Dis. 2008 Nov 13;3:29. doi: 10.1186/1750-1172-3-29 (PMC2596089; doi:10.1186/1750-1172-3-29)
Supplement: Additional file 3 — Adjusted odds ratios for potential risk factors for parietal pleural plaques, DPT and lung fibrosis in the asbestos-exposed subjects according to an ordinal regression analysis. [file 1750-1172-3-29-S3.doc]

### Additional file 3. Adjusted odds ratios for potential risk factors for parietal pleural plaques, DPT and lung fibrosis in the asbestos-exposed subjects according to an ordinal regression analysis

| Variables | Odds Ratio* | 95% CI | p Value |
| --- | --- | --- | --- |
| Pleural plaques |  |  |  |
| RPF | 1.20 | 0.33-4.32 | 0.782 |
| Age at the HRCT scanning | 1.00 | 0.92-1.07 | 0.901 |
| Smoking in pack-years | 1.00 | 0.98-1.03 | 0.872 |
| DPT grade | 1.32 | 0.75-2.33 | 0.330 |
| Lung fibrosis grade | 3.78 | 1.52-9.43 | 0.004 |
| DPT |  |  |  |
| RPF | 3.06 | 0.81-11.56 | 0.099 |
| Age at the HRCT scanning | 1.08 | 0.99-1.18 | 0.070 |
| Smoking in pack-years | 1.02 | 0.99-1.05 | 0.226 |
| Pleural plaque grade | 1.32 | 0.66-2.66 | 0.437 |
| Lung fibrosis grade | 1.01 | 0.45-2.26 | 0.990 |
| Lung fibrosis |  |  |  |
| RPF | 1.29 | 0.25-6.63 | 0.759 |
| Age at the HRCT scanning | 1.07 | 0.97-1.18 | 0.159 |
| Smoking in pack-years | 0.99 | 0.95-1.02 | 0.472 |
| DPT grade | 1.13 | 0.61-2.10 | 0.688 |
| Pleural plaques grade | 2.73 | 1.10-6.78 | 0.030 |

CI, confidence interval; DPT, diffuse pleural thickening; HRCT, high-resolution computed tomography; RPF, retroperitoneal fibrosis.

* Odds ratio indicates the risk associated with a one-level increase in the variable (e.g., risk associated with a 1-year increase at the age the HRCT scanning took place).
